# The Effects of Congruence and Incongruence in Parental Co-Parenting on Adolescents’ Depression: Using Polynomial Regression with Response Surface Analysis

**DOI:** 10.3390/bs16030448

**Published:** 2026-03-18

**Authors:** Xiaoqing Wang, Ruisen Chen, Panqin Ye, Furong Lu

**Affiliations:** School of Education Science, Shanxi University, Taiyuan 030006, China; wxq821@sxu.edu.cn (X.W.); chenruisen@sxu.edu.cn (R.C.); yepanqinzi@sxu.edu.cn (P.Y.)

**Keywords:** co-parenting, depression, self-esteem, response surface analysis

## Abstract

This study explores the influence of congruence and incongruence in father–mother co-parenting on adolescent depression, as well as the mediating effect of self-esteem. A total of 1389 adolescents completed questionnaires assessing their levels of depression and self-esteem, while their fathers and mothers correspondingly reported on their own co-parenting behaviors using the Parental Co-parenting Scale in this cross-sectional study. Dates were analyzed using LPA, RSA, and mediation consecutively. The results show that: (1) We identified three distinct co-parenting profiles: positive parental co-parenting, negative parental co-parenting, and mixed parental co-parenting. (2) In cases of congruent parental co-parenting, high positive parental co-parenting was associated with lower adolescent depression, whereas high negative parental co-parenting was linked to higher depression, and the difference manifests in different forms among boys and girls. Girls showed nonlinear changes in depression while boys exhibited linear trends. (3) In cases of incongruence in parental co-parenting, mothers’ co-parenting exerted a stronger influence on boys’ depression, while girls were not affected by mothers’ and fathers’ discrepancies. (4) Self-esteem mediated the relationship between parental co-parenting (in)congruence and depression across both genders. This study provides evidence for the mechanism through which parental coparenting influences adolescent depression and offers a basis for future interventions targeting adolescent depression.

## 1. Introduction

In recent years, the mental health issues of adolescents have drawn widespread concern from families and society. Studies indicate that the detection rate of depressive symptoms among Chinese adolescents range from 24% to 28%, showing a rising trend year by year (G. L. Yu, 2022). Depression during adolescence can significantly impair academic performance and school adjustment. According to the “2024 Adolescent Mental Health and Academic Status Survey” released by the Chinese Academy of Sciences, students with higher mental health risks are more likely to report not wanting to attend school, exhibit poorer academic resilience, and have weaker academic self-efficacy (Sun et al., 2025). Moreover, such adverse effects may extend into adulthood, leading to psychosocial problems such as anxiety and poor social adaptation. Depression during adolescence not only significantly impairs academic performance and social adaptability but also predicts a series of future psychological issues, including anxiety and suicide, causing damage to individual physical and mental health (X. C. Zhang et al., 2025; McLeod et al., 2016; Weersing et al., 2017; Becker & Correll, 2020). Therefore, exploring the risk factors and mechanisms of adolescent depression is of great significance for promoting individual physical and mental health.

The family is a crucial environment for the growth and development of adolescents; parenting styles are one of the direct factors that influence adolescent mental health (Y. Q. Ma et al., 2022). As a key family environmental factor, parenting styles have received increasing attention regarding their impact on adolescent depression. The degree to which fathers and mothers are able to support each other in child-rearing practices is crucial for the healthy development of adolescents (Xie et al., 2021). These collaborative parenting activities between adults who share the responsibility of raising a child are referred to as “co-parenting” (C. Liu et al., 2014; C. Liu & Wu, 2015).

However, existing studies have focused on individual dimensions while overlooking the potentially heterogeneous configurations of co-parenting. As Mandara (2003) noted, co-parenting constitutes a multidimensional and integrated whole, wherein the same parent may simultaneously exhibit multiple parenting styles in varying combinations. In recent years, a great deal of empirical studies have adopted person-centered approaches to identify the substantive typologies of parenting styles, rather than focusing on specific dimensions. Latent profile analysis (LPA), a commonly used person-centered method, aims to better capture inter-individual heterogeneity (Laursen & Hoff, 2006; S. H. Liu et al., 2023; Zhan et al., 2023). Consequently, a growing consensus suggests that psychological research should first identify the underlying patterns of parenting styles before examining their correlations and outcomes (Berge et al., 2010; Hoeve et al., 2009).

Indeed, the family functions as a complex ecosystem within which the parenting behaviors of mothers and fathers are interactive rather than independent (Cox & Paley, 1997). On one hand, individuals tend to select partners whose behavioral patterns resemble their own (Lyons et al., 2020); this assortative mating contributes to congruence in parenting practices. On the other hand, to maintain functional balance and integrity within the family, parents often adopt complementary parenting roles—such as the traditional pattern of a “strict father and nurturing mother” or “nurturing father and strict mother”—thereby exhibiting incongruent parenting styles (Dwairy, 2010). These highlight the necessity of examining both consistency and incongruence in parental co-parenting within family systems research. Generally speaking, mothers play the central role of carer in child-rearing (Zou et al., 2019). whereas fathers tend to serve a more instrumental role (Y. Xu et al., 2006). However, with the development of society, contemporary parenting beliefs and involvement have changed (J. J. Xu et al., 2023). Fathers are now devoting more time and effort to raising children (X. Li, 2020), with the importance of the fathers’ role in the family becoming increasingly apparent (Hou et al., 2022). As fathers become more involved in child-rearing, congruent co-parenting between both parents can reduce the risk of adolescent depression. According to the “Consistency-Adaptation Hypothesis” proposed by the Diverging Operations Triad Model (Delos Reyes & Ohannessian, 2016), the higher levels of co-parenting in child-rearing, the fewer adolescents’ problem behaviors have, such as good academic performance and lower levels of depression (Lu et al., 2019; Wen et al., 2023). Co-parenting in child-rearing activities can reduce the risk of depression in adolescents. Studies show that spousal support boosts both the parental responsibility and sensitivity of mothers and fathers, which contributes to a warmer family environment (Y. H. Li et al., 2024). When fathers cooperate with mothers in parenting activities, they can effectively share the burden of mothers’ child-rearing responsibilities (Gao et al., 2020) and foster a stable family environment for adolescents to grow up (T. T. Liu et al., 2021). A positive family environment provides adolescents with secure emotional support, reducing their emotional stress and risk of depression (J. J. Li et al., 2018; W. J. Liu et al., 2012; C. F. Chen et al., 2021).

Conversely, if one of the parents fails to cooperate with the other parent’s parenting activities, in other words, parental incongruence in co-parenting leads to an imbalance in parental involvement, creating a conflicted family atmosphere. This results in adolescents lacking stable emotional responses, which is harmful to their physical and mental health development. According to the “Consistency-Adaptation Hypothesis” of the Diverging Operations Triad Model, when parents have incongruent parenting styles, adolescents have more problem behaviors and poorer emotional skills, such as aggressive behaviors and a risk of depression (Yin et al., 2022). Recent studies highlight that when parental co-parenting is incongruent, adolescents cannot feel supportive co-parenting, leading to an increased risk of depression. Among adolescents experiencing negative family support, 29.2% exhibit a risk of depression (Fu et al., 2024). Yang et al. (2021) and G. Z. Zhang et al. (2021) found that incongruence in co-parenting impairs adolescents’ self-regulation, creates a negative family environment, and increases adolescents’ depression (Yang et al., 2021; G. Z. Zhang et al., 2021). Therefore, incongruent parenting activities create a conflict family atmosphere, depriving adolescents of stable emotional responses and clear rule-based guidance, which in turn leads to adolescents experiencing a sense of being abandoned and causing negative emotions such as depression and anxiety (Schoppe et al., 2001).

However, the impact of incongruence in parental co-parenting on adolescent depression may vary. Higher levels of congruence in fathers and mothers may have different effects on depression, particularly among adolescents of different genders. Studies have found by Sher-Censor et al. (2011) that compared to fathers, mothers’ positive parenting behaviors are more strongly associated with adolescents’ emotional well-being. Research by Tang et al. (2024) also found that mothers’ positive parenting activities had a greater influence on adolescents’ internalizing problem behaviors than fathers’, but fathers’ positive parenting activities had a greater impact on adolescent externalizing problem behaviors than mothers’ (Tang et al., 2024). Additionally, the impact of incongruence in parental co-parenting between fathers and mothers may differ between boys and girls. According to the theory of gendered family and cognitive development, parenting styles also have different influences on the development of boys and girls (Endendijk et al., 2018). Adolescents are more influenced by parents of the same gender. Fathers’ involvement in co-parenting provides boys with emotional security and reduces depression in boys (Zheng et al., 2025). Fathers’ involvement in the family can significantly influence boys’ emotions and control abilities (Nie & Lu, 2014). Huston (1983) noted that parents bear greater responsibility for the socialization of same-gender offspring. L. Xu et al. (2018) also found that children are more susceptible to the influence of same-gender parents. The father–son relationship significantly predicts internalizing and externalizing problem behaviors among boys, while mother–daughter relationships significantly predict such problems in girls. However, some research also indicates that fathers have a greater influence on daughters. For instance, when fathers are more involved in parenting activities, daughters tend to achieve higher academic standards, while this effect is not observed in sons (Paul & Usha, 2021). It may be inferred that higher levels of congruence in parental co-parenting on adolescents’ depression have different effects on depression in boys and girls.

Based on this, the present study aims to first identify the patent profiles of parental co-parenting using Latent Profile Analysis (LPA) and then employ Response Surface Analysis (RSA) to examine the effects of parental co-parenting congruence and incongruence on adolescent depression. The objective is to reveal how the mechanism of co-parenting affects adolescent depression, providing empirical evidence for family interventions and mental health promotion.

Self-esteem refers to individuals’ overall positive evaluation of themselves, reflecting their perception and attitude towards their own abilities and value (Leary & Baumeister, 2000). It has a significant impact on individual emotions and problem behaviors (Hao & Cui, 2007; Jia et al., 2016). Parenting styles play a primary role and have a significant impact on children’s self-esteem. Recent studies show that congruence in parental co-parenting fosters a harmonious family atmosphere, promoting children’s positive self-perception as well as enhanced self-esteem (J. J. Chen et al., 2014; G. Q. Liu et al., 2020). Conversely, incongruence in parental co-parenting can damage adolescents’ self-esteem (C. Liu et al., 2017). According to the vulnerability model of depression, low self-esteem plays a risk role for depression (D. L. Li et al., 2019). Adolescents’ self-esteem levels are negatively correlated with depression (Zhou et al., 2021; S. Yu & Liu, 2020). According to the theory of intrinsic relations in the host, individuals form internal psychological structures through interactions with significant others, which subsequently shape their emotions and behaviors in adulthood. Adolescents who feel high levels of co-parenting and experience a secure emotional environment may internalize a sense of “valuable self”, thereby contributing to the development of adolescent self-awareness. This process reduces the risk of psychological problems such as depression (Wang et al., 2025).

Consequently, adolescents living in environments with low levels of co-parenting may experience negative self-concepts and self-esteem due to experiencing more emotional neglect, ultimately increasing the risk of depression.

Based on the above, this study proposes the following hypothesis:

**H1.**  *There exist multiple latent profiles of parental co-parenting styles.*

**H2.**  *When parental co-parenting is congruent, different patterns of congruent co-parenting exert different effects on adolescent depression, and the difference manifests in different forms among boys and girls.*

**H3.**  *When parental co-parenting is incongruent, different patterns of incongruent co-parenting exert different effects on adolescent depression, and the difference manifests in different forms among boys and girls.*

**H4.**  *Self-esteem mediates the relationship between parental (in)congruence in co-parenting and adolescent depression.*

## 2. Materials and Methods

### 2.1. Participants and Procedures

Convenient cluster sampling was used in this study to distribute 1389 questionnaires and conduct surveys in Shanxi Province, China, and Henan Province, China. The questionnaires of adolescents matched the father and mother from the same family (52.8% boys; age 8–16, M = 12). The initial sample consisted of 1562 adolescents and their parents. A total of 173 questionnaires were excluded due to missing data, failure to match parent–adolescent dyads, or lack of informed consent, yielding a final valid sample of 1389 participants (effective response rate: 88.9%).

During the data analysis phase, block variables reflecting the parental congruence and incongruence in co-parenting were constructed using polynomial regression coefficients. These block variables were then employed as independent variables to conduct mediation effect tests. This study employed a cross-sectional design, with data collected at a single time point from all participants.

### 2.2. Measures

#### 2.2.1. Depression

Depression in adolescents was assessed using the Center for Epidemiologic Studies Depression Scale (CES-D), developed by Radloff (1977). In this scale, the depression dimension comprises twenty items rated on a 4-point scale ranging from 1 (Not at all) to 4 (Most of the time). The higher the score, the more severe the depression. In this study, this scale had good internal consistency and reliability (Cronbach’s α = 0.95; NFI = 0.95, CFI = 0.91, RMSEA = 0.06).

#### 2.2.2. Co-Parenting

The parental co-parenting questionnaire employed the Coparenting Scale developed by Chinese scholars C. Liu et al. (2014) was adopted in this study. The co-parenting dimension comprises eighteen items, utilizing a Likert 7-point scale (1 = never, 7 = always) comprising four dimensions: unity, consistency, conflict, and disparagement. For mothers’ co-parenting, Cronbach’s α for the unity, consistency, conflict, and disparagement dimensions were 0.88, 0.84, 0.81, and 0.78, respectively. This scale had good internal consistency and reliability (NFI = 0.91, CFI = 0.92, RMSEA = 0.07). For fathers’ co-parenting, the internal consistency coefficients for unity, consistency, conflict, and disparagement dimensions were 0.88, 0.86, 0.80, and 0.87, respectively. Confirmatory factor analysis indicated good internal consistency and reliability (NFI = 0.91, CFI = 0.92, RMSEA = 0.07).

#### 2.2.3. Self-Esteem

The self-esteem questionnaire employed the Self-Esteem Scale (SES) developed by Rosenberg (1965). In this scale, the self-esteem dimension comprises ten items utilizes a 4-point Likert scale 1 (highly consistent) to 4 (not consistent at all), with higher total scores indicating greater self-esteem. In this study, this scale had good internal consistency and reliability (Cronbach’s α = 0.94, NFI = 0.90, CFI = 0.93, RMSEA = 0.06).

### 2.3. Procedure

This study has been approved by the university ethics committee. Participants were briefly informed of the purpose and precautions of the study before completing the questionnaire. With the school’s consent, the tests were conducted by trained psychology graduate students. The students’ and parents’ responses were collected immediately after completing the questionnaire.

### 2.4. Materials and Methods

Firstly, the latent profile analysis to investigate types of parental co-parenting with R 4.5.2. Afterwards, the descriptive statistics and response surface analysis (RSA) were applied to analyze the effects of (in)congruence in positive and negative parental co-parenting, respectively, on adolescent depression, and the mediating effects on adolescent depression were calculated with SPSS 27.0. and Mplus 8.

## 3. Results

### 3.1. Common Method Bias Test

Using Harman’s one-way test, the results showed that there were nine factors with eigenvalues greater than one, and the cumulative variation explained by the first factor was 23.03%, which was less than the critical criterion of 40%, so this study did not have a serious problem of common method bias.

### 3.2. Descriptive Statistics and Correlation Analysis

**Figure 1:**
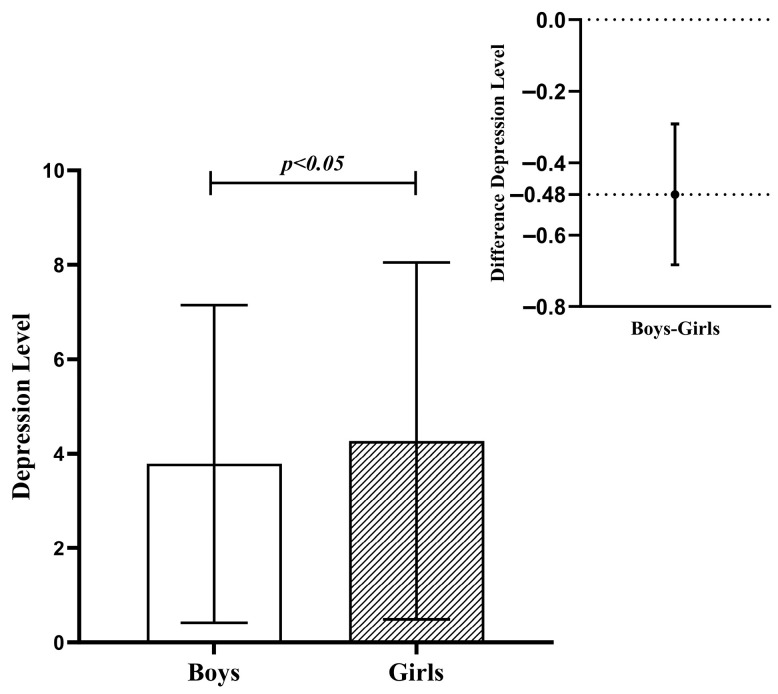
Gender differences in depression.

Table 1 presents the descriptive statistics for all variables. Mothers’ and fathers’ unity and consistency were found to be positively correlated with adolescent self-esteem and negatively correlated with adolescent depression. However, mothers’ and fathers’ conflict and disparagement were found to be negatively correlated with adolescent self-esteem and negatively correlated with adolescent depression. The adolescent self-esteem was negatively correlated with adolescent depression. In addition, the independent samples t-test revealed a significant difference in depression scores between boys and girls (see Table 2); girls’ levels of depression are significantly higher than boys’ [t(1389) = −2.47, *p* < 0.05] (see Figure 1).

### 3.3. A Latent Profile Analysis of Parental Co-Parenting Activities

Latent Profile Analysis (LPA) was used to derive categorical latent variables, which represent classes of individuals who share similar profiles (Lanza et al., 2003). Co-parenting styles include multiple dimensions, and the manifestations of fathers’ and mothers’ behaviors on positive and negative dimensions exist in complex configurations that traditional variable-centered approaches struggle to capture. Moreover, as a person-centered method, Latent Profile Analysis (LPA) can identify heterogeneous latent subgroups within the sample, which accords with the reality of mutual influence between both parents in family systems theory. Therefore, this study first conducted LPA scores of four dimensions: fathers’ and mothers’ unity, consistency, conflict, and disparagement.

**Figure 2:**
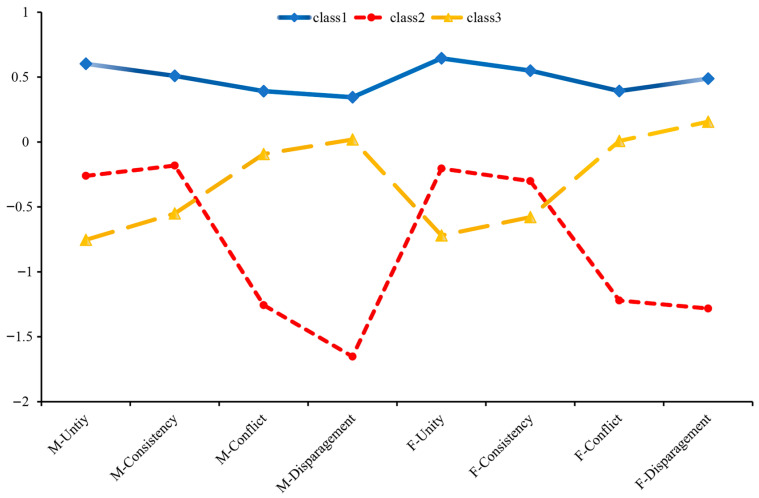
Mean Score Distribution of Standardized Dimensions for 3 Potential Categories of Co-Parenting. Profile 1 = “Mixed Parental Co-Parenting”; Profile 2 = “Positive Parental Co-Parenting”; Profile 3 = “Negative Parental Co-Parenting”.

LPA was conducted to identify the best-fitting profile solution. Table 3 showed the results for the profile solutions that ranged from one to four classes. The entropy value for all models exceeds 0.8, indicating that each model demonstrates good classification accuracy. From 1-profile to 4-profile, the AIC, BIC, and aBIC values progressively decrease, indicating a significant improvement in goodness-of-fit. Nevertheless, in the category probability of 4-profile, categories account for less than 10% of the sample. Moreover, the Entropy for the 3-profile solution was higher than the 0.81 observed for the 4-profile, indicating slightly better distinction among the three classes. Additionally, ambiguous categories were identified within the 4-profile structure, leading to the discarding of the four-category solution. Considering the overall fit of the model, the 3-profile solution was the best-fitting model for the data (see Figure 2). The results of the post-test are shown in Table 4. Based on the LPA, Profile 1 (42.3% of the sample) was characterized by “higher positive co-parenting and higher negative co-parenting” compared to Profile 2 and Profile 3, labeling “Mixed Parental Co-Parenting”. Profile 2 (31.7% of the sample) indicated higher scores on unity and consistency but lower scores on conflict and disparagement compared to the others, labeling “Positive Parental Co-Parenting”. Profile 3 (26.0% of the sample) exhibited higher scores on conflict and disparagement but lower scores on unity and consistency, labeling “Negative Parental Co-Parenting”. The four potential categories of co-parenting have different dimensional characteristics, and the scores of the specific standardized dimensions are shown in Table 4.

The results revealed three latent types of parental co-parenting: “Positive Parental Co-parenting” (Profile 2), “Negative Parental Co-parenting” (Profile 3), and the “Mixed Parental Co-parenting” (Profile 1). Furthermore, the findings indicate that parental co-parenting behaviors manifest not only in congruent patterns (e.g., Profile 2 and Profile 3) but also in complex combinations (e.g., Profile 1). Consequently, we have reason to speculate that within the family, there may be incongruence in co-parenting types where mothers adopt positive co-parenting, coexist with fathers adopt negative co-parenting, or mothers adopt negative co-parenting, coexist with fathers adopt positive co-parenting. Therefore, the following study further employed Response Surface Analysis (RSA) to investigate the nonlinear effects of both congruence and incongruence in positive and negative coparenting on adolescent depression, thereby revealing the dynamic mechanisms underlying parental coparenting.

### 3.4. Response Surface Analysis Method Based on Polynomial Regression

Response Surface Analysis (RSA), based on polynomial regression, models the complex relationship between independent and dependent variables, while response surface analysis builds upon this by constructing a three-dimensional surface plot to visualize the relationships between two independent variables and the dependent variable. It further utilizes the lines of congruence and incongruence to reflect the degree of alignment between the two predictors (Shanock et al., 2010). This approach addresses several limitations of the traditional difference-score method (i.e., computing the absolute difference between two variables), including spurious correlations, reduced reliability, and difficulties in interpreting regression coefficients (Song et al., 2024). It also allows for a more thorough investigation of the unique effects of two predictor variables, as well as the magnitude of effects of their congruence and incongruence on the outcome variable (Ostroff et al., 2005), thus providing a more precise and comprehensive analytical tool for scientific research.

After controlling for the age of the adolescents, stepwise regressions. This study employs a Response Surface Analysis method based on polynomial regression. First of all, father and mother co-parenting were centered around the pooled grand mean to reduce multicollinearity before calculating the second-order terms (Shanock et al., 2010). Adolescent depression (AD) was the dependent variable, centered on mothers’ co-parenting (M), centered fathers’ co-parenting (F), mothers’ co-parenting squared (M^2^), mothers’ co-parenting times fathers’ co-parenting (M × F), and fathers’ co-parenting squared (F^2^). b_0_ represents the intercept, the level of AD when both M and F are zero; b_1_ and b_2_ are the coefficient of the linear terms M and F, respectively, representing the linear relationship between M and F themselves and AD; b_3_ and b_5_ are the coefficients of the quadratic terms M^2^ and F^2^, respectively, reflecting the non-linear relationship between M and F with AD; b_4_ is the coefficient of the interaction term MF, reflecting the interaction between M and F on AD; e represents the random error. The equation applied was as follows (to simplify the equation, control variables were omitted):

AD = b_0_ + b_1_M + b_2_F + b_3_M^2^ + b_4_MF + b_5_F^2^ + e (1)

Calculate the response surface curve parameters a_1_ to a_4_. The effect of the independent variable (parental positive or negative co-parenting) on the congruence of the dependent variable (adolescent depression) is primarily reflected through the significance of the slopes and curvatures of the congruent line (M = F) and the incongruent line (M = −F). The congruent line (M = F) indicates a congruence effect of father and mother co-parenting levels that are matched. Its slope parameter a_1_ = b_1_ + b_2_. The significance of a_1_ reflects the impact of co-parenting levels on the dependent variable. Its curvature parameter a_2_ = b_3_ + b_4_ + b_5_ is used to test whether this congruent relationship exhibits non-linear characteristics. The incongruent line (M = −F) represents that parental positive or negative co-parenting levels are different. Its slope parameter is a_3_ = b_1_ − b_2_. The significance of a_3_ indicates the direction of the incongruence (i.e., “high father positive co-parenting − low mother positive co-parenting” versus “low father positive co-parenting − high mother positive co-parenting” or “high father negative co-parenting − low mother negative co-parenting” versus “low father negative co-parenting − high mother negative co-parenting”) influences the dependent variable. If significant, this suggests different effects of the incongruence on adolescent depression. Its curvature parameter a_4_ = b_3_ − b_4_ + b_5_. The significance of a_4_ reflects the impact of the degree of incongruence on the dependent variable.

### 3.5. The (In)Congruence Effect of Parental Co-Parenting on Adolescents’ Depression

A cluster analysis was conducted on fathers’ and mothers’ positive co-parenting, using a 0.5 standard deviation threshold. In the samples of girls and boys, the percentages of cases with M > F were 28.9% and 26.1%, respectively; those with M < F were 29.3% and 27.8%, respectively; and those with M = F accounted for 41.8% and 46.1%, respectively. The relatively even distribution of cases across these categories renders the data suitable for response surface analysis. The regression analysis indicates that the R^2^ values were 0.32 (*p* < 0.001) and 0.36 (*p* < 0.001), respectively, in Table 5. Firstly, to examine the congruence effect (M = F), Table 5 presents the results of polynomial regression and response surface analysis about the relationship between parental co-parenting and adolescent depression.

**Figure 3:**
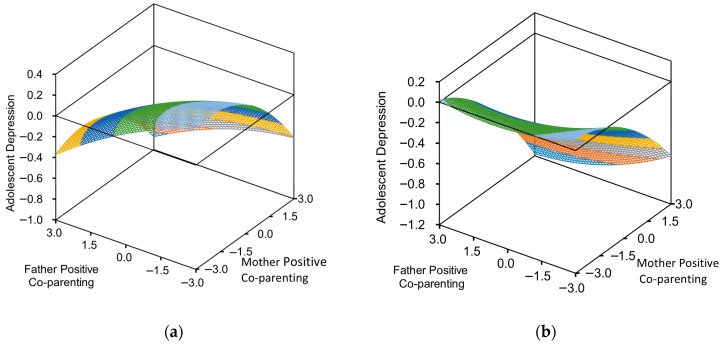
(**a**) Positive parental co-parenting response surface curve diagram for depression levels in girls; (**b**) positive parental co-parenting response surface curve diagram for depression levels in boys.

Within the dimension of positive co-parenting, the slope a_1_ (M = F) of the positive parental co-parenting consistency line is negatively significant (a_1_ = −0.20, *p* < 0.001), (a_1_ = −0.18, *p* < 0.001). It indicates that under conditions of positive congruent co-parenting, adolescent depression levels were lower when both parents exhibited high (rather than low) levels of positive co-parenting. Furthermore, in the girls’ sample, the curvature coefficient (a_2_) was negatively significant (a_2_ = −0.12, *p* < 0.001), indicating a nonlinear trend: as the level of positive congruent co-parenting decreased, girls’ depression levels increased at an accelerating rate. This suggests a marginally increasing effect on this relationship. The curvature parameter a_2_ for boys’ depression levels (a_2_ = −0.03, *p* = 0.47) was not significant, indicating that the linear trend influence of congruence in positive parental co-parenting on boys’ depression (see Figure 3a,b).

Incongruence (M = −F) examines the impact of high levels of fathers’ positive co-parenting with low levels of mothers’ positive co-parenting (i.e., “high father − low mother”) and low levels of fathers’ positive co-parenting with high levels of mothers’ positive co-parenting (i.e., “low father − high mother”) on adolescent depression. Analysis of the boys and girls, respectively. For girls, the slope coefficient a_3_ was not significant (a_3_ = 0.01, *p* = 0.92), indicating that differences in positive parental co-parenting had no significant effect on depression levels. For boys, however, when mothers’ positive co-parenting levels were higher than fathers’ (i.e., “high mother − low father”), their depression levels were lower (a_3_ = −0.15, *p* < 0.05). This suggests that mothers play a more significant role in boys (see Figure 3a,b).

In the dimension of negative parental co-parenting, the proportions of cases with M > F were 27.1% for parents of girls and 24.0% for parents of boys, respectively; those with M < F were 27.4% and 24.2%, respectively; and those with M = F accounted for 45.5% and 51.8%, respectively. The distribution of cases across these comparison groups was relatively balanced, and the data were suitable for response surface analysis. The results in Table 6 of the regression analysis indicated that, for the negative co-parenting dimension, the models for parents of girls and boys explained a significant portion of the variance, with R^2^ values of 0.22 (*p* < 0.05) and 0.28 (*p* < 0.05), respectively, demonstrating good overall explanatory power. Table 6 presents the detailed results of the polynomial regression and response surface analysis examining the relationship between negative parental co-parenting and adolescent depression. The R^2^ in Model 1 is just 0.12, but this is common in psychosocial surface models.

**Figure 4:**
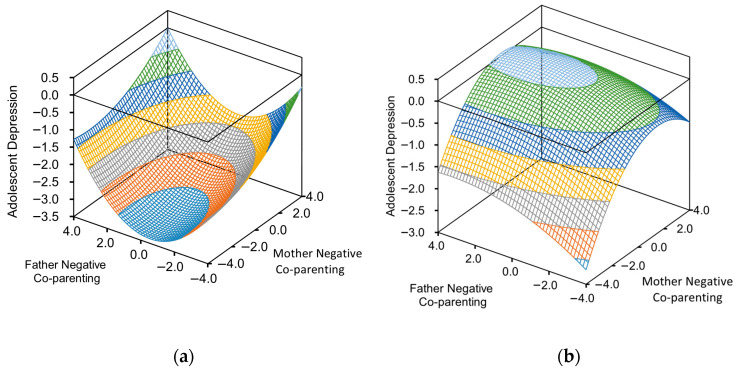
(**a**) Negative parental co-parenting response surface curve diagram for depression levels in girls; (**b**) negative parental co-parenting response surface curve diagram for depression levels in boys.

In the dimension of negative co-parenting, the slope of the congruence line (M = F) was positive in both girls and boys (a_1_ = 0.12, *p* < 0.05) (a_1_ = 0.14, *p* < 0.001). This indicates that under conditions of high congruent co-parenting (both father and mother exhibit negative co-parenting), adolescent depression levels were higher compared to conditions of low congruent co-parenting. In the sample of girls, the curvature a_2_ was significantly positive (a_2_ = 0.08, *p* < 0.05), indicating a non-linear trend in the effect for girls, specifically showing that as the congruence of negative co-parenting increases, the rate of increase in girls’ depression levels accelerates, reflecting a marginal increasing effect. In contrast, within the boys’ sample, the curvature a_2_ was not significant (a_2_ = −0.02, *p* = 0.37). This suggests a linear trend in the effect of negative parental congruent co-parenting on depression among boys (see Figure 4a,b).

Regarding the incongruence condition (M = −F), to examine the effects of “high father negative co-parenting − low mother negative co-parenting” (i.e., “high father − low mother”) versus “low father negative co-parenting − high mother negative co-parenting” (i.e., “low father − high mother”) on adolescent depression, analyses were conducted separately for girls and boys. For girls, slope a_3_ was not significant (a_3_ = 0.03, *p* = 0.29), indicating that the discrepancy between parents’ negative co-parenting did not significantly affect their depression levels. For boys, however, when parents exhibited negative co-parenting, higher levels of mothers’ negative co-parenting compared to fathers’ negative co-parenting (i.e., “high mother − low father”) were associated with higher depression levels in boys (a_3_ = 0.18, *p* < 0.001). This finding also suggests that the mother plays a more significant role in the parenting of boys (see Figure 4a,b).

### 3.6. Testing for the Moderated (In)Congruence Effects on Adolescents’ Depression

**Figure 5:**
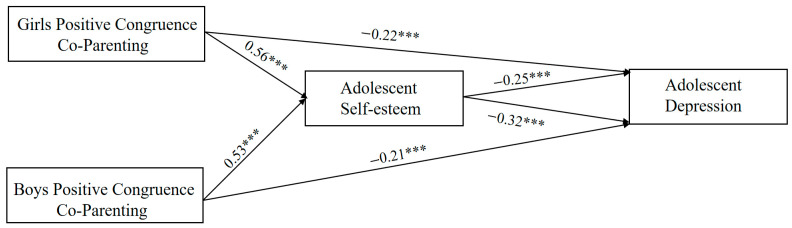
Pathways of positive parental co-parenting influence adolescent depression in girls and boys. Note. *** *p* < 0.001.

Block variable analysis was employed to examine the mediating effect of adolescent self-esteem. Combine either fathers’ positive co-parenting and mothers’ positive co-parenting or fathers’ positive co-parenting and mothers’ positive co-parenting into a block variable. Using this block variable as the independent variable, adolescent self-esteem as the mediating variable, and adolescent depression as the dependent variable, conduct separate analyses for positive parental co-parenting and negative parental co-parenting (Figure 5). All variables were standardized before data analysis, and the mediating effects were tested using the PROCESS 4.2 with 5000 bootstrap samples to assess the significance of the indirect effects.

The analysis results are shown in Table 7. For both boys and girls, the block variables of mothers’ and fathers’ positive co-parenting have a significantly positive influence on adolescent self-esteem (β_girls_ = 0.56, t_girls_ = 4.87, *p*_girls_ < 0.001) (β_boys_ = 0.53, t_boys_ = 4.62, *p*_boys_ < 0.001). The first half of the mediating pathway holds true. Self-esteem demonstrated a negatively significant association with depression (β_girls_ = −0.25, t_girls_ = −3.14, *p*_girls_ < 0.001) (β_boys_ = −0.32, t_boys_ = −3.39, *p*_boys_ < 0.001).

**Figure 6:**
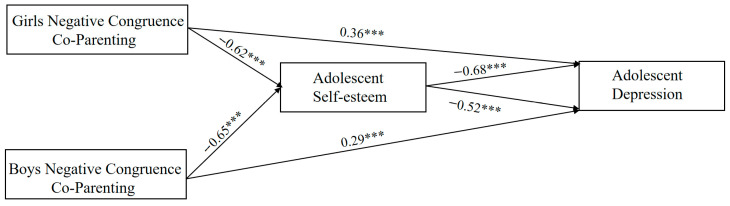
Pathways of negative parental co-parenting influence adolescent depression in girls and boys. Note. *** *p* < 0.001.

The analysis results are shown in Table 8. For both boys and girls, the block variables of mothers’ and fathers’ negative co-parenting have a negatively significant influence on adolescents’ self-esteem (β_girls_ = −0.62, t_girls_ = −6.93, *p*_girls_ < 0.001; β_boys_ = −0.65, t_boys_ = −7.20, *p*_boys_ < 0.001). The first half of the mediating pathway holds true; adolescent self-esteem has a negatively significant influence on adolescent depression (β_girls_ = −0.68, t_girls_ = −7.76, *p*_girls_ < 0.001; β_boys_ = −0.52, t_boys_ = −4.54, *p*_boys_ < 0.001). The last half of the mediating pathway holds true. This indicates that block variables mediate the relationship between adolescent depression and self-esteem (see Figure 6).

## 4. Discussion

### 4.1. Latent Classes and Characteristics of Parental Co-Parenting

Co-parenting is a multidimensional parent–child interaction process that is not limited to a single, uniform pattern. Parents may display distinct co-parenting patterns across different contexts. Based on Latent Profile Analysis and the distribution characteristics of scores across co-parenting dimensions, the study identified three latent profiles of parental co-parenting: Positive Co-Parenting, Negative Co-Parenting, and Mixed Co-Parenting. The results indicated that Mixed Co-Parenting accounted for the largest proportion of the sample, characterized by both unity and conflict, whereas the Negative Co-Parenting class was the least prevalent. These findings are largely consistent with the reality of parenting styles in Chinese families. In contemporary China, many families are dual-earner households with fewer children, and co-parenting is not a stable interaction pattern but rather a dynamic process shaped by parenting goals, educational beliefs, and emotional states. In most ordinary families, parents demonstrate cooperation based on shared parenting goals, while simultaneously experiencing conflicts arising from divergent educational beliefs and life stress. The mixed pattern of co-parenting is generally consistent with previous empirical evidence (W. J. Liu et al., 2012; Wu et al., 2016).

In summary, the study employed a person-centered approach to identify three latent classes of co-parenting, capturing the diversity of parenting styles present in Chinese families. On one hand, regarding the different dimensions of co-parenting, the relationship between the traditional dichotomous classification—positive and negative co-parenting—is not static; instead, positive and negative co-parenting may coexist. On the other hand, considering both fathers and mothers, parenting styles within the family system may be either homogeneous or heterogeneous between parents. Therefore, it is necessary and meaningful to examine the combined parenting patterns of fathers and mothers.

### 4.2. The Relationship Between Congruence and Incongruence in Parental Co-Parenting on Adolescents’ Depression

Based on the theoretical framework of Diverging Operations Triad Mode (Delos Reyes & Ohannessian, 2016), this study employed polynomial regression and response surface analysis to examine the effects of both positive parental co-parenting congruence—incongruence and negative parental co-parenting congruence—incongruence on adolescent depression, alongside the mediating effect of self-esteem within this relationship. Research has found that girls’ levels of depression are significantly higher than boys’, which may be attributed to parents adopting different parental activities towards children of different genders. For instance, Verhoeven et al. (2010) found that parents were more inclined to use corporal punishment to discipline boys, whereas they were less likely to use corporal punishment on girls, instead resorting more frequently to emotional punishment (Verhoeven et al., 2010; Kwang et al., 2013; Leaper & Brown, 2018). On the one hand, during the process of growth, girls tend to place greater emphasis on intimate relationships (Peterson, 2000). They are more likely to become emotionally involved in parents’ conflicts, feeling a more intense sense of unease and loneliness (Flook, 2011; S. Y. Hu et al., 2019; Y. Q. Hu et al., 2023). On the other hand, compared to males, psychological and physiological stressors related to social rejection can elicit a greater HPA axis response in females. This heightened physiological reactivity to stressors induced by negative parental activities provides a biological basis for the development of depression (Cai et al., 2016; Goel et al., 2014). When parents adopt negative parental activities, girls are more likely than boys to adopt rumination as a response strategy, whereas boys may release stress through externalizing problem behavior, resulting in higher levels of depression among girls than boys (Dong et al., 2020).

The present study found that when parents exhibited high-congruent positive co-parenting (i.e., both father and mother exhibited high positive co-parenting), adolescents had significantly lower levels of depression than in the low-congruent positive co-parenting group (i.e., both father and mother exhibited low positive co-parenting). Similarly, under the condition of negative congruent co-parenting, adolescents demonstrated higher levels of depression in the high-congruent negative co-parenting group (i.e., both father and mother exhibited high negative co-parenting) than in the low-congruent negative co-parenting group (i.e., both father and mother exhibited low negative co-parenting), thereby supporting Hypothesis 1. This is because parents’ co-parenting activities can develop complementary support systems, reducing parents’ conflicts, and providing adolescents with a stable family environment that enhances their sense of security (M. J. Liu et al., 2022). When both parents actively engage in their adolescents’ lives and establish positive interactions, this dual support system more effectively alleviates adolescents’ negative emotions by providing emotional encouragement and emotional regulation skills, thereby reducing their risk of depression (Deng et al., 2013). When positive congruence in parental co-parenting is low (i.e., both father and mother exhibited low positive co-parenting), adolescents have higher levels of depression. This finding confirms the Co-Parenting Ecological Model, which states that when parents fail to engage in supportive parenting behaviors, it results in frequent co-parenting conflict. Such conflicts not only diminish the father’s parental involvement but also cause the mother’s negative emotions, leading to employing negative parental activities towards the child (Whitlock et al., 2018; Su et al., 2019). In addition, negative mothers’ parenting activities are closely associated with adolescent depression (Guo et al., 2023).

Furthermore, the effects of co-parenting congruence on adolescent depression varied by gender. For girls, depression exhibited a marginally increasing effect as parental congruence in positive co-parenting decreased. In other words, girls’ depression levels accelerated as positive parental co-parenting congruence declined. This may be because girls are more adept than boys at perceiving emotional information (Hall, 1978), rely more heavily on family emotional support, and are more sensitive to family dynamics (Mandara & Murray, 2000; Yuan et al., 2010). For girls, conflicts in parenting activities may heighten their awareness of instability within the family system, leading to a sharp increase in depression. In contrast, for boys, depression increased linearly with declining parental congruence in positive co-parenting, consistent with the chronic stress and support deprivation model. When parental positive co-parenting congruence is low, the family support system is weaker, progressively undermining boys’ psychological support systems and consuming their internal resources (McHale et al., 2000). Consequently, their depression showed linear progression.

When positive parental co-parenting is incongruent, the difference in parental co-parenting activities does not significantly influence depression among girls. This validates Gottman’s (1993) “Balancing Theory”, which means that if one of the parents exhibits negative parental activities, the other parent will establish a secure emotional base. The expression of positive emotions and behaviors will gradually counterbalance the risks associated with negative parenting practices (Ju et al., 2020). Among boys, when mothers’ levels of positive parental co-parenting are higher than fathers’ (“low fathers’ positive co-parenting − high mothers’ positive co-parenting”), boys’ depression is lower. This finding supports the irreplaceable role of the mother as the primary secure base. High levels of mothers’ positive co-parenting foster emotional regulation skills and stable internal working models (F. Q. Zhao et al., 2022; X. J. Liu & Li, 2022), effectively buffering the risks associated with low fathers’ co-parenting. On the one hand, within contemporary family education, mothers are the primary educators and nurturers of adolescents (Cui et al., 2012; S. Ma et al., 2021), with their parental activities exerting a greater influence on adolescents (Baptista et al., 2017). On the other hand, influenced by traditional Chinese culture, mothers tend to adopt more supportive co-parenting behaviors towards sons, who are of a different gender to themselves (McHale, 1995).

### 4.3. The Mediating Effect of Self-Esteem

The results demonstrated that, for both girls and boys, the block variables representing positive parental co-parenting congruence and negative parental co-parenting congruence not only directly influenced adolescent depression but also exerted indirect effects through self-esteem. These findings suggest, to some extent, that individual self-esteem serves as a critical mechanism through which family rearing environments shape adolescent depression, while also providing empirical support for the vulnerability model of depression within the family context. This model posits that low self-esteem is a stable personality risk factor for depression. Within the family environment, when parents lack supportive co-parenting and emotional response, adolescents struggle to obtain positive feedback regarding self-worth, thereby inhibiting the development of self-esteem. Individuals with low self-esteem are more likely to have negative self-attributions, rendering them more vulnerable to depressive affect when confronted with stress (Riina & McHale, 2014; H. Y. Zhao et al., 2024). Meanwhile, this study reveals that modest gender differences in these mediating pathways. Specifically, the indirect effect of parental co-parenting on depression via self-esteem was slightly lower among boys than among girls. This discrepancy may be attributable to gender-specific characteristics of self-esteem development and patterns of emotional expression. Such subtle differences may reflect gender disparities in adolescents’ sensitivity to the family emotional atmosphere. Specifically, girls’ self-esteem development is more susceptible to family emotional bonding, and the family environment shaped by parental co-parenting exerts a greater impact on girls’ self-esteem.

Overall, the mediating effect of self-esteem validates the core proposition of object relations theory. As a critical family interaction experience in early adolescence, parental co-parenting shapes adolescents’ inherent psychological structures through internalization processes, thereby influencing their emotional adjustment before adulthood. The level of congruence in parental co-parenting informs the formation of adolescents’ self-concept: positive co-parenting fosters adolescent mental health, whereas negative co-parenting damages adolescent depression via the mediating role of self-esteem.

Finally, the three co-parenting profiles suggest that the three co-parenting profiles identified in this study offer a screening framework for school counselors to identify adolescents at risk for depression. The mediating role of self-esteem suggests that interventions targeting self-concept may buffer the adverse effects of incongruent co-parenting. Gender-specific findings further support the need for tailored family-based prevention strategies according to adolescent gender.

### 4.4. Strengths, Limitations, and Future Research

This study focused on adolescents in Chinese family contexts. Latent Profile Analysis (LPA) was used to identify latent classes of parental co-parenting. Polynomial regression with Response Surface Analysis (RSA) was applied to examine the effects of co-parenting congruence and incongruence on adolescent depression, as well as gender differences. Block variable analysis was conducted to test the mediating role of self-esteem. The mechanism linking parental co-parenting to adolescent depression was systematically revealed. However, the present study has several limitations. Firstly, this study is a cross-sectional study, making it difficult to examine the longitudinal relationship between parental co-parenting and adolescent depression, and the above results cannot help us draw causal relationships. Future studies could explore the use of longitudinal data for this purpose. Secondly, this study utilized a questionnaire method, which may have introduced social approval bias. Future research on aggressive behavior could benefit from using experimental methods. Additionally, the data sample of this study is only from China, so the subject group is not large enough to be generalized to other regions or countries. Cross-cultural comparisons between China and other regions with distinct ethnic compositions may further elucidate how cultural norms modulate the co-parenting–adolescent depression linkage. Finally, the present sample covers a relatively wide age range. It is important to note that early to middle adolescence is a critical period of rapid physical and psychological development. Future research should employ longitudinal follow-up designs or conduct stratified analyses by developmental stage (e.g., childhood vs. adolescence) to thoroughly examine whether the impact of parental co-parenting on adolescent depression changes as individuals develop, thereby identifying more precise, age-specific targets for intervention.

## 5. Conclusions

First, three latent classes of parental co-parenting were identified: Mixed Parental Co-Parenting, Positive Parental Co-Parenting, and Negative Parental Co-Parenting. The mixed type accounted for the largest proportion (42.3%), while negative co-parenting accounted for the least (26.1%).

Second, parental co-parenting congruence affects adolescent depression, with gender differences and dimensional differences. For positive co-parenting, high congruent positive co-parenting significantly reduced adolescent depression, whereas low congruent positive co-parenting increased depression. For girls, depression showed a marginally increasing nonlinear trend as positive co-parenting congruence decreased; for boys, depression increased linearly. For negative co-parenting, high congruent negative co-parenting significantly elevated depression. For girls, depression showed a marginally increasing nonlinear trend as negative co-parenting congruence increased; for boys, depression again increased linearly.

Third, the impact of co-parenting incongruence on adolescent depression differed significantly by gender, with similar gender patterns across positive and negative dimensions. For girls, incongruence in both positive and negative co-parenting showed no significant effect on depression, suggesting that positive parenting from one parent can buffer the risks of negative parenting from the other, supporting the balance theory. For boys, the “high mothers’ co-parenting- low fathers’ co-parenting” pattern in positive co-parenting significantly reduced depression, whereas the same pattern in negative co-parenting significantly increased depression. This supports the central role of mothers in boys’ upbringing, indicating that maternal parenting has an irreplaceable influence on boys’ depression.

Fourth, self-esteem mediated the relationship between parental co-parenting congruence and adolescent depression. Congruent positive co-parenting positively predicted self-esteem, which in turn negatively predicted depression. Congruent negative co-parenting negatively predicted self-esteem, which also negatively predicted depression.

This study advances our understanding of the complex dynamics of mothers’ and fathers’ co-parenting families and the necessity of simultaneously examining both mothers’ and fathers’ parenting behaviors. Practically, interventions aimed at reducing adolescent depression risk should prioritize enhancing positive co-parenting congruence and improving adolescent self-esteem. These findings provide precise directions for depression prevention and intervention efforts targeting adolescents.

## Figures and Tables

**Table 1:** Descriptive statistics and correlation analysis of variables.

Variable	M ± SD	1	2	3	4	5	6	7	8	9	10	11
1. Age	12.16 ± 1.99	1										
2. Mother—Unity	4.44 ± 1.15	0.02	1									
3. Mother—Consistency	4.65 ± 1.24	0.04	0.14 ***	1								
4. Mother—Conflict	2.28 ± 1.04	0.09 ***	−0.11 ***	−0.62 ***	1							
5. Mother—Disparagement	1.59 ± 0.83	0.04	−0.32 ***	−0.27 ***	0.12 ***	1						
6. Father—Unity	4.21 ± 1.21	0.17 ***	0.39 ***	0.12 ***	−0.10 ***	−0.54 ***	1					
7. Father—Consistency	4.54 ± 1.28	0.09 ***	0.04	0.44 ***	−0.33 ***	−0.23 ***	0.16 ***	1				
8. Father—Conflict	2.29 ± 1.02	0.03	−0.07 **	−0.33 ***	0.32 ***	0.15 ***	−0.17 ***	−0.63 ***	1			
9. Father—Disparagement	1.56 ± 0.88	0.01	−0.11 ***	−0.08 **	0.12 ***	0.16 ***	−0.15 ***	−0.02	0.05	1		
10. Adolescent Self-Esteem	2.93 ± 0.50	−0.12 ***	0.05	0.08 **	−0.08 **	−0.12 ***	0.11 ***	0.03	−0.04	−0.69 ***	1	
11. Adolescent Depression	0.20 ± 0.18	−0.21 ***	−0.14 ***	−0.03	0.06 **	0.17 ***	−0.16 ***	−0.06 **	0.02	0.14 ***	−0.10 ***	1

Note. *** *p* < 0.001, ** *p* < 0.01.

**Table 2:** Gender differences in adolescent depression levels.

**Adolescent Depression**	**Gender**	**Cases**	**M (SD)**	**t**	***p***	**Differential Comparison**
	Boys	655	3.79 (3.36)	−2.47	0.01	Boys < Girls
	Girls	734	4.27 (3.78)			

**Table 3:** Model fit indices for the latent profile analysis solutions (*n* = 1389).

Number of Categories	AIC	BIC	a BIC	Entropy	VLMR (*p*)	a LMR (*p*)	BLRT (*p*)	Category Probability
1	33,601.76	33,686.54	33,636.77					
2	32,026.68	32,159.16	32,055.32	0.90	<0.001	<0.001	<0.001	16.03/83.96
3	31,126.06	31,306.23	31,238.86	0.88	<0.001	<0.001	<0.001	42.28/31.66/26.04
4	30,935.13	31,162.99	31,043.19	0.83	0.017	0.028	<0.001	39.49/37.24/13.58/9.68

Note. AIC = Akaike Information Criterion; BIC = Bayesian Information Criterion; a BIC = Sample Size-Adjusted Bayesian Information Criterion; VLMR (*p*): Vuong-Lo-Mendell-Rubin Likelihood Ratio Test; a LMR (*p*): Adjusted Lo-Mendell-Rubin Likelihood Ratio Test; BLRT (*p*) = Bootstrap Likelihood Ratio Test.

**Table 4:** Comparison of Mean Scores of Each Dimension Item Across Latent Classes of Different Parental Co-Parenting.

Dimension	Profile 1	Profile 2	Profile 3	F	Post-Examination
Mother-Unity	0.60	−0.26	−0.75	1950.23 ***	P1 > P2 > P3
Mother-Consistency	0.51	−0.18	−0.55	1880.47 ***	P1 > P2 > P3
Mother-Conflict	0.39	−1.26	−0.09	860.19 ***	P1 > P3 > P2
Mother-Disparagement	0.35	−1.65	0.02	1580.62 ***	P1 > P3 > P2
Father-Unity	0.65	−0.20	−0.72	1920.35 ***	P1 > P2 > P3
Father-Consistency	0.55	−0.30	−0.58	1890.71 ***	P1 > P2 > P3
Father-Conflict	0.39	−1.22	0.01	875.28 ***	P1 > P3 > P2
Father-Disparagement	0.49	−1.28	0.16	1610.94 ***	P1 > P3 > P2

Note. *** *p* < 0.001.

**Table 5:** Response surface regression of positive parental co-parenting with girls and boys.

Variable	Model 1		Model 2	
	β	t	β	t
(Constant)	−0.03	−0.55	−0.15	−3.83
M	−0.11	−2.15 **	−0.11	−2.81 ***
F	−0.10	−2.01 **	−0.13	−3.28 ***
M^2^	−0.03	−0.83	−0.12	−3.02 **
MF	0.08	−1.82 *	−0.07	−1.64
F^2^	−0.02	−0.61	−0.10	−2.47 **
Congruence Line (LOC)				
Slope: a_1_ (b_1_ + b_2_)	−0.20	−4.61 ***	−0.18	−3.96 ***
curvature: a_2_ (b_3_ + b_4_ + b_5_)	−0.12	−2.75 ***	−0.02	−0.53
Incongruence Line (LOIC)				
Slope: a_3_ (b_1_ − b_2_)	−0.01	−0.08	−0.17	−3.70 ***
Curvature: a_4_ (b_3_ − b_4_ + b_5_)	−0.04	−0.41	0.05	0.71
Spindle Match LOC				
a_5_ (b_3_ − b_5_)	−0.13		−0.33	
R^2^	0.32 **		0.36 ***	
F	37.43		43.58	
ΔR^2^	0.06		0.08	

Note. Model 1: the relationship between positive parental co-parenting and depression in girls; Model 2: the relationship between positive parental co-parenting and depression in boys. Note. *** *p* < 0.001, ** *p* < 0.01, * *p* < 0.05.

**Table 6:** Response surface regression of negative parental co-parenting with girls and boys.

Variable	Model 1		Model 2	
	β	t	β	t
(Constant)	−0.42	−1.82	−0.03	0.61
M	0.13	2.47 **	0.17	3.24 ***
F	−0.06	−1.92 *	0.09	2.43 *
M^2^	−0.04	−1.00	−0.07	−2.20 **
MF	0.08	2.35 **	−0.01	0.47
F^2^	0.10	2.61 ***	−0.02	−1.01
Congruence Line (LOC)				
Slope: a_1_ (b_1_ + b_2_)	0.12	2.36 **	0.14	2.65 ***
curvature: a_2_ (b_3_ + b_4_ + b_5_)	0.08	2.34 **	−0.02	−0.48
Incongruence Line (LOIC)				
Slope: a_3_ (b_1_ − b_2_)	0.03	0.10	0.18	3.99 ***
Curvature: a_4_ (b_3_ − b_4_ + b_5_)	0.36	8.64 ***	−0.11	1.96 *
Spindle Match LOC				
a_5_ (b_3_ − b_5_)	−0.05		−0.05	
R^2^	0.12 **		0.28 **	
F	12.43		30.13	
ΔR^2^	0.01		0.03	

Note. Model 1: The relationship between parental co-parenting and depression in girls; Model 2: the relationship between parental co-parenting and depression in boys. Note. *** *p* < 0.001, ** *p* < 0.01, * *p* < 0.05.

**Table 7:** Standard effect sizes and 95% CI for each pathway in the mediating model in positive co-parenting.

Pathway	Standardized Effect Size	95% CI
Girls’ Positive Congruence Co-Parenting Overall Effect	−0.36 ***	(−0.73, −0.15)
Girls’ Positive Congruence Co-Parenting→Adolescent Depression	−0.22 ***	(−0.52, −0.11)
Girls’ Positive Congruence Co-parenting→Self-Esteem→Adolescent Depression	−0.14 ***	(−0.36, −0.05)
Boys’ Positive Congruence Co-Parenting Overall Effect	−0.38 ***	(−0.82, −0.03)
Boys’ Positive Congruence Co-Parenting→Adolescent Depression	−0.21 ***	(−0.49, −0.18)
Boys’ Positive Congruence Co-Parenting→Self-Esteem→Adolescent Depression	−0.17 **	(−0.33, −0.02)

Note. *** *p* < 0.001, ** *p* < 0.01.

**Table 8:** Standard effect sizes and 95% CI for each pathway in the mediating model in negative co-parenting.

Pathway	Standardized Effect Size	95% CI
Girls’ Negative Congruence Co-Parenting Overall Effect	0.78 ***	(0.45, 1.12)
Girls’ Negative Congruence Co-Parenting→Adolescent Depression	0.36 ***	(0.09, 0.75)
Girls’ Negative Congruence Co-parenting→Self-Esteem→Adolescent Depression	0.42 ***	(0.12, 0.95)
Boys’ Negative Congruence Co-Parenting Overall Effect	0.63 ***	(0.32, 1.24)
Boys’ Negative Congruence Co-Parenting→Adolescent Depression	0.29 ***	(0.08, 0.54)
Boys’ Negative Congruence Co-Parenting→Self-Esteem→Adolescent Depression	0.34 ***	(0.13, 0.68)

Note. *** *p* < 0.001.

## Data Availability

The data that support the findings of this study are available on request from the corresponding author. The data are not publicly available due to privacy or ethical restrictions.
